# Draft Genome Sequences of *Dermacoccus nishinomiyaensis* Strains UCD-KPL2534 and UCD-KPL2528 Isolated from an Indoor Track Facility

**DOI:** 10.1128/genomeA.01652-16

**Published:** 2017-02-23

**Authors:** Brian A. Klein, Katherine P. Lemon, Prasad Gajare, Guillaume Jospin, Jonathan A. Eisen, David A. Coil

**Affiliations:** a Department of Microbiology, The Forsyth Institute, Cambridge, Massachusetts, USA; b Department of Oral Medicine, Infection and Immunity, Harvard School of Dental Medicine, Boston, Massachusetts, USA; c Division of Infectious Diseases, Boston Children’s Hospital, Harvard Medical School, Boston, Massachusetts, USA; d University of California Davis Genome Center, Davis, California, USA; e Department of Evolution and Ecology and Department of Medical Microbiology and Immunology, University of California Davis, Davis, California, USA

## Abstract

We present here the draft genome sequences of *Dermacoccus nishinomiyaensis* strains UCD-KPL2534 and UCD-KPL2528, which were isolated at an indoor track facility in Medford, MA, USA (42.409716, -71.115169) from an exit door handle and settle dust, respectively. The genome assemblies contain 3,088,111 bp in 58 contigs and 3,162,381 bp in 100 contigs, respectively.

## GENOME ANNOUNCEMENT

Members of the genus *Dermacoccus* have been previously isolated from deep-ocean sediment (1, 2), coral (3, 4), tap water (5), humans (6), insects (7–9), and cured meat (10). *Dermacoccus* spp. are characterized as Gram-positive, nonmotile, and aerobic cocci that commonly produce orange pigment (6, 11, 12). Interest in *Dermacoccus* spp. has focused on phenazine derivative production for potential applications as dyestuffs or antioxidant compounds (13–15). *Dermacoccus* spp. are rarely pathogens to humans, with only one report of a central venous catheter infection and two reports of *Dermacoccus* spp. potentially involved in polymicrobial infections (16–18). Recently, Chng and colleagues suggested that *Dermacoccus* spp. might antagonize *Staphylococcus* spp. during flares of atopic dermatitis, a potentially beneficial role (19).

We isolated *Dermacoccus nishinomiyaensis* UCD-KPL2534 from a metal door handle and *Dermacoccus nishinomiyaensis* UCD-KPL2528 from settle dust of an indoor track facility in Medford, MA, as part of a project to produce reference genomes for microbes resident in the built environment (20). A nylon-flocked swab (COPAN) dipped in sterile buffer (0.1 M NaCl and 0.1% Tween 80) was rubbed on the surfaces, inoculated onto selective brain heart infusion agar containing fosfomycin (20 μg/ml), and incubated aerobically at 37°C for 5 days. Two small circular pigmented colonies were selected for analysis. Genomic DNA for whole-genome sequencing was extracted using the MasterPure complete DNA and RNA purification kit (Epicentre).

Illumina paired-end libraries were generated using a Nextera DNA sample prep kit (Illumina). We selected 600- to 900-bp fragments using a Pippin Prep (Sage Science) and sequenced the resulting libraries on an Illumina MiSeq, with a read length of 300 bp, which produced 1,573,606 (UCD-KPL2534) and 4,093,434 (UCD-KPL2528) paired-end reads. Quality trimming and error correction of the reads resulted in 1,429,177 and 3,703,158 high-quality reads using the A5-miseq assembly pipeline (version 05/22/2015) (21). The assembly for strain UCD-KPL2534 contained 58 scaffolds (minimum, 604 bp; maximum, 591,826 bp; *N*_50_, 171,024 bp). The assembly for strain UCD-KPL2528 contained 100 scaffolds (minimum, 1,037 bp; maximum, 523,322 bp; *N*_50_, 119,501 bp). The final assemblies both have a G+C content of 69.1% and error-corrected coverage estimates of 57- and 133-fold for UCD-KPL2534 and UCD-KPL2528, respectively. We assessed genome completeness with PhyloSift and CheckM; all PhyloSift marker genes were present, and CheckM reported 100% completeness with less than 1% contamination estimations for both isolates (22, 23).

The genomes were annotated using the RAST server (default settings, 14 April 2016) (24). *D. nishinomiyaensis* strains UCD-KPL2528 and UCD-KPL2534 both contain 2,892 predicted coding sequences (CDSs). Thirty-one proteins differ based strictly on predicted presence. Additionally, two partial phages are predicted in strain UCD-KPL2528 but not strain UCD-KPL2534; neither strain has a predicted clustered regularly interspaced short palindromic repeat (CRISPR) system (25, 26). Both genomes harbor clusters encoding carotenoid biosynthesis with greatest similarity to *Dermacoccus* sp. Ellin185. *D. nishinomiyaensis* UCD-KPL2534 also has two putative biosynthetic clusters encoding antifungal macrolide/macrocyclic molecules that strain UCD-KPL2528 and other published *Dermacoccus* spp. lack (27).

We assigned a putative species designation of *Dermacoccus nishinomiyaensis* to each isolate using both PhyloPhlAn and 16S-rRNA-gene-based phylogeny of *Dermacoccus* spp. generated from sequences in the Ribosomal Database Project (28, 29) (https://doi.org/10.6084/m9.figshare.4284344.v1).

### Accession number(s).

This whole-genome shotgun project is deposited at DDBJ/ENA/GenBank under the accession numbers MQVT00000000 and MQVU00000000. The versions described in this paper are MQVT01000000 and MQVU01000000, respectively.
